# Comparison of ustekinumab, infliximab and combination therapy in moderately to severely active ulcerative colitis – a study protocol of a randomized, multicenter, head-to-head COMBO-UC trial

**DOI:** 10.3389/fmed.2024.1458998

**Published:** 2024-09-19

**Authors:** Renata Talar-Wojnarowska, Adam Fabisiak, Hubert Zatorski, Katarzyna Płoszka, Katarzyna Błaziak, Wojciech Fendler, Grażyna Rydzewska, Ewa Małecka-Wojciesko, Piotr Eder

**Affiliations:** ^1^Department of Digestive Tract Diseases, Medical University of Lodz, Lodz, Poland; ^2^Department of Biostatistics and Translational Medicine, Medical University of Lodz, Lodz, Poland; ^3^Clinical Department of Internal Medicine and Gastroenterology with Inflammatory Bowel Disease Unit, National Medical Institute of the Ministry of the Interior and Administration, Warsaw, Poland; ^4^Department of Gastroenterology, Dietetics and Internal Medicine, Poznań University of Medical Sciences, Poznań, Poland

**Keywords:** inflammatory bowel diseases, ulcerative colitis, dual biological therapy, infliximab, ustekinumab

## Abstract

**Background:**

Ulcerative colitis (UC) is a chronic inflammatory bowel disease with a complex etiology that affects the large intestine. Characterized by chronic, bloody diarrhea, UC can lead to severe complications, including an increased risk of colorectal cancer. Despite advancements in conservative treatment, including biologics like anti-TNF agents and ustekinumab (UST), many patients do not achieve full remission. Dual targeted therapy (DTT) combining infliximab (IFX) and UST is a promising approach to improve treatment outcomes.

**Methods:**

This prospective, randomized, multicenter, head-to-head controlled trial will evaluate the efficacy and safety of UST, IFX, and combination therapy (UST + IFX) in 172 patients with moderate to severe active UC across eight gastroenterology centers in Poland. The study includes a 14–16 week remission induction period followed by a 52-week maintenance phase. Patients will be randomly assigned to one of three treatment arms: IFX monotherapy, UST monotherapy, or IFX + UST combination therapy. Primary endpoint is clinical and endoscopic remission post-induction. Secondary endpoints include clinical response, biochemical remission, histological remission, and quality of life assessments using the Inflammatory Bowel Diseases Questionnaire and 36-Item Short Form Survey. Safety will be monitored through adverse event and serious adverse event reporting.

**Discussion:**

This trial aims to determine whether combining IFX and UST can achieve higher remission rates and better long-term outcomes compared to monotherapy. The results could provide crucial insights into the optimal use of biologic agents in UC treatment, potentially establishing DTT as a standard therapy. The study’s design, including extensive follow-up and robust endpoint measures, will contribute to understanding the therapeutic potential and safety profile of this combination therapy.

## 1 Introduction

Ulcerative colitis (UC), belonging to the group of inflammatory bowel diseases (IBD), is an incurable condition of the large intestine with frequent systemic, extraintestinal manifestations (1–3). The etiology of UC is unknown. However, it seems that in genetically predisposed individuals, in the presence of some hypothesized environmental factors, poorly defined triggering factors (like intestinal dysbiosis) induce and maintain inflammatory lesions in the bowel wall (4–7). As a result, chronic, bloody diarrhea is the main symptom of the disease, but in long-term irreversible damage of the intestine can lead to its serious malfunction, increasing the risk of complications like colorectal cancer (1–3).

UC mainly affects young adults and children, but currently the disease incidence is especially increasing among older adults and/or elderly people (1). The prognosis in UC is difficult to assess, however it should be emphasized that even one-third of patients need to undergo surgical treatment (colectomy) at different stages of the disease. Due to these data, together with the fact of increasing prevalence of UC reported in many industrialized and developing countries, UC is more and more frequently considered as a serious medical, social, and economical problem (1, 2, 8, 9).

Recent years have brought significant improvement in non-surgical therapeutic options in UC (1–3, 10). The introduction of anti-tumor necrosis factor (anti-TNF) antibodies more than 20 years ago opened the new era of targeted treatment in IBD. Subsequent approval of other biologic agents, like vedolizumab (VDZ), ustekinumab (UST) or mirikizumab, together with the newest group of orally administered small molecule drugs (Janus kinases inhibitors and sphingosine-1-phosphate receptor modulators), have further broadened the therapeutic armamentarium (1, 10). Unfortunately, although the efficacy of individual agents was shown to be significantly better than placebo in different clinical trials and the overall clinical benefit from introducing these new treatment options is unquestionable, long-term observations still provide some disappointing conclusions. Namely, it has been shown that none of the advanced therapeutic molecules is able to induce full disease remission in more than half of the patients (10, 11). Moreover, it is still a matter of debate whether the increasing number of treatment options can be directly translated into changing the long-term natural history of UC (10–13).

That is why, to break through this therapeutic ceiling, several treatment concepts have been proposed (11). Dual targeted therapy (DTT), being a combination treatment with two parallelly used advanced molecules, is one of the most promising approaches (14). To this date, however, there has been only one randomized controlled trial published, showing the benefits of DTT with guselkumab (GUS, anti-interleukin-23 antibody) and golimumab (GOL, anti-TNF agent) over monotherapy with GUS or GOL in moderately-to-severely active UC (15). At the same time, there is a growing number of case report series available, confirming the benefits of DTT, without any additional safety concerns (14).

These promising results allowed entering a new era of combined treatment in IBD. Nevertheless, considering the paucity of data, there are still many questions which have to be answered before considering DTT to become a standard therapy in UC or Crohn’s disease (CD). One of the most important issues is how to combine different modes of action to achieve the highest efficacy and lowest rates of adverse events. The results of clinical trials and multiple real-life studies confirm the crucial role of therapeutic TNF inhibition in IBD (16). Indeed, infliximab (IFX), being the most frequently used TNF-inhibiting molecule, is characterized by a rapid onset of action and potent anti-inflammatory effect. At the same time, high rates of secondary loss of response still belong to the most important limitations of anti-TNF treatment in IBD (16–18). Considering the data suggesting the crucial role of IL-23 in promoting the refractoriness of immune cells (mainly T cells and macrophages) to TNF inhibition, combining IFX with another monoclonal antibody interfering with IL-23-dependent pathways seems to be a promising therapeutic approach in IBD (19). That is why we proposed conducting a clinical trial verifying the usefulness of DTT with IFX – a TNF inhibitor, and UST - a monoclonal antibody directed against the p40 subunit of IL-23 and IL-12 in UC, which is another biologic agent with confirmed efficacy and safety both in clinical trials, as well as in real-life cohorts (20, 21).

The study is funded by the Medical Research Agency (2022/ABM/03/00013). The protocol was approved by the European Medicines Agency under EU CT number 2023–506452–25-00. The trial will be conducted in accordance with the ethical principles set out in the Declaration of Helsinki and the guidelines on Good Clinical Practice. The study was registered in the clinicaltrials.gov registry (ClinicalTrials.gov identifier: NCT06453317).

## 2 Methods and analysis

### 2.1 Aim of the study

The main goal of the study is to compare the efficacy and safety of UST, IFX and combination therapy (UST + IFX) in patients with moderate to severe active UC.

### 2.2 Study design

**Figure 1:**
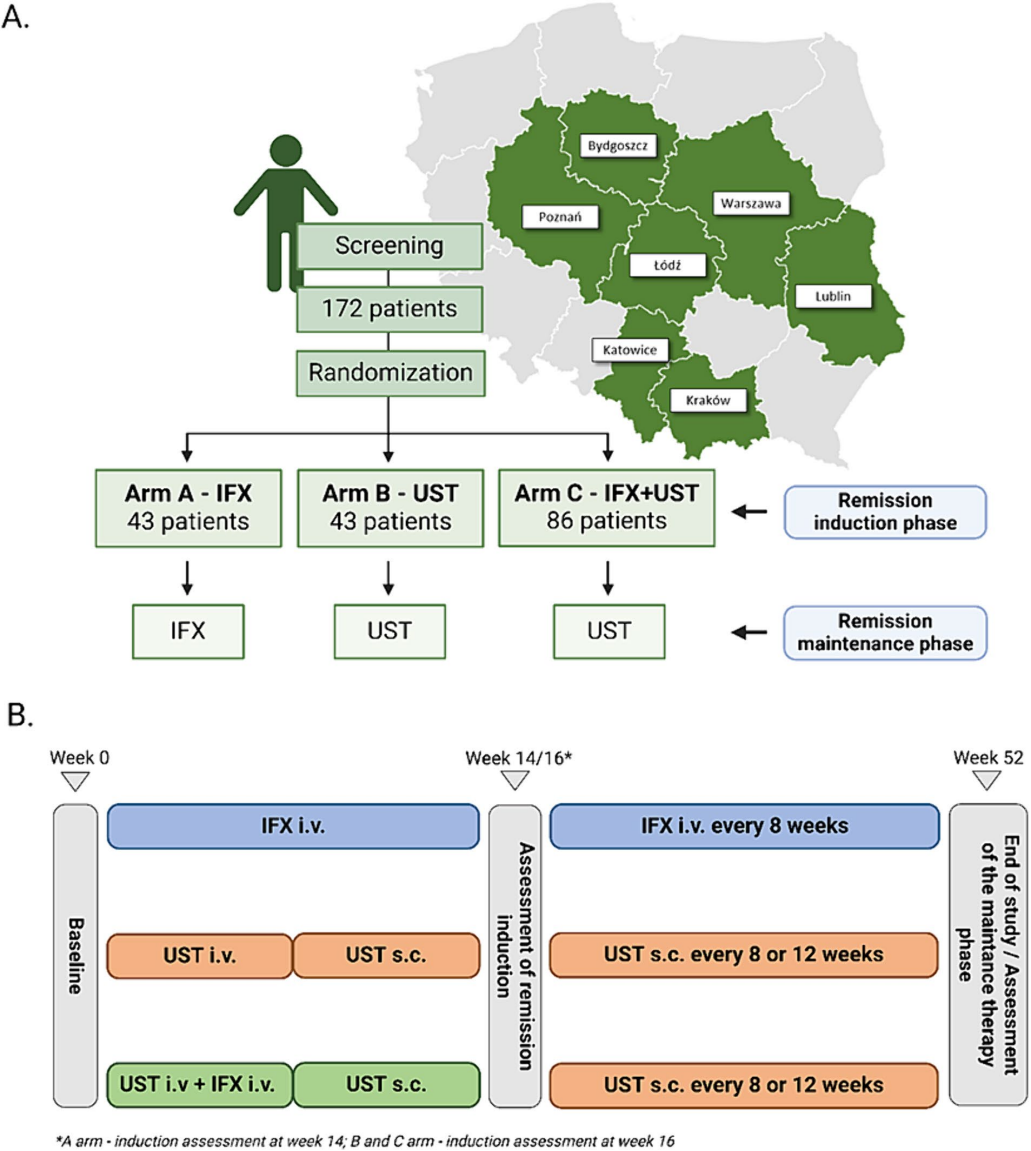
Allocation of patients to treatment arms and map of centers recruited for the study **(A)**. Basic scheme of treatment arms **(B)**.

This study will be a prospective, randomized, multicenter, head-to-head, controlled trial. One hundred seventy-two patients with moderate to severe active UC from the eight tertiary gastroenterology centers in Poland will be enrolled in the study. The study protocol assumes a 14–16 weeks remission induction period and then a remission maintenance period lasting until week 52 in all arms. After completing the full intervention period of 52 weeks, patients will be followed up at week 56. Figure 1A shows the allocation of patients to treatment arms.

Our treatment schedule follows the guidelines of the national program for treating patients with UC B.55, as published by the National Health Fund in Poland. Therefore, we implemented a 16-week induction period for patients receiving UST. This approach is supported by the UNIFI long-term extension study, where 79.3% of the Week 16 delayed responders who entered the long-term extension sustained symptomatic remission at Week 92 (22).

The patients will be informed of the purposes and conditions of the study and will be asked to sign the relevant informed consent form. The patients will also be informed of the option to refuse and withdraw their consent at any time, without stating the reason or suffering any consequences, and without losing the right to receive further care in the centers involved. The patients will receive no compensation for their participation in the study.

### 2.3 Intervention

After recruitment, the participants will receive IFX or UST according to the study protocol, which summary is presented in Figure 1B. Induction of remission will last for 14 weeks in A-arm and 16 weeks in B-and C-arm. Patients who will achieve clinical response defined as reduction in PRO-2 score > 50% or a reduction in total Mayo score > 3 points will be qualified to maintenance.

The remission induction phase in A-arm will include 3 doses of IFX in week 0, 2 and 6, whereas subjects entering maintenance receive IFX every 8 weeks. Patients in A-arm will receive IFX in dose 5 mg/kg i.v. for the whole induction and maintenance phase. Participants in the C arm will receive IFX only in the induction phase at a dose of 5 mg/kg i.v. in weeks 0, 2, and 6.

Subjects in B-arm and C-arm will receive one dose of UST i.v at week 0. depending on their weight following one dose of 90 mg of UST s.c. at week 8 as induction therapy. UC patients with a weight ≤ 55 kg will receive 260 mg of UST, those with a weight between 55 and 85 kg will receive 390 mg, and those with a weight ≥ 85 kg will receive 520 mg i.v. Patients who achieve clinical response after induction therapy will receive further doses of UST s.c. in the maintenance phase. The dose of UST in maintenance therapy will be 90 mg s.c. irrespective of patients’ weight.

UST product characteristics allow the administration of UST every 8 or 12 weeks in maintenance therapy in UC patients. Thus, depending on the activity of UC in participants interval between drug doses will be to Investigator’s choice.

### 2.4 Patients

The study will include 172 adult patients with established diagnosis of UC. Patients will be qualified for trial according to the inclusion and exclusion criteria of drug program B.55 published by National Health Found to be eligible for the trial, subjects must meet all of the inclusion criteria and none of the exclusion criteria, as stated below.

### 2.5 Inclusion criteria

- Subjects who are voluntarily able to give informed consent
- Subjects who can participate in all aspects of this clinical study
- Males and females aged from ≥ 18 to ≤ 80 years, at screening visit
- Diagnosis of moderate to severe UC established at least 3 months before screening by clinical and endoscopic evidence, corroborated by a histopathology report and confirmed by the investigator
- Moderately to severely active UC as determined by a total Mayo score of 7 to 12 confirmed by a central reader within 28 days prior to randomization
- Demonstrated an inadequate response to, loss of response, or intolerance to standard treatment consisting of corticosteroids and/or 6-mercaptopurine or azathioprine
- Patients receiving 5-ASA derivatives, corticosteroids, or immunosuppressive agents may be included in the study on condition that will receive stable doses of aforementioned drugs for 14 days before randomization (i.e., the dosage of each medication must remain unchanged for 14 consecutive days before the randomization visit)
- At screening, females of childbearing potential must be non-pregnant and non-lactating or females should be of non-childbearing potential
- Female patients of childbearing potential, with a fertile male sexual partner, must use highly effective contraception from screening until 15 weeks after the last dose of UST and 6 months after the last dose of IFX study drug
- Female subjects must not donate ovarian oocytes until 6 months after the last dose of the study drug
- Negative urine or serological pregnancy test at the screening visit

### 2.6 Exclusion criteria

- Previous exposure to IFX or UST or any other investigational IFX or UST-containing product
- Has a history of hypersensitivity or allergies to the ingredients of IFX or UST formulations
- Unstable angina
- Moderate or severe heart failure (defined as New York Heart Association Class III or IV)
- Chronic respiratory failure
- History of any major neurological disorders including stroke, multiple sclerosis, brain tumor, or neurodegenerative disease that in the opinion of the investigation would cofound the study results
- Current or recent history of alcohol dependence or illicit drug abuse
- PML diagnosis positive PML subjective symptom checklist prior to the randomization
- Chronic hepatitis B or C infection. Patients with positive viral serology at screening for infection with hepatitis B (HBV) or hepatitis C virus (HCV) may be eligible if the polymerase chain reaction test is negative, and the patients receive standard of-care antiviral prophylaxis (if applicable)
- Known severe chronic kidney failure
- Known severe chronic liver failure
- Known active or latent tuberculosis
- Has received total parenteral or enteral nutrition
- Ongoing HIV infection
- History of indeterminate colitis or ischemic colitis
- History of diverticulitis 60 days before randomization
- Pregnancy or breastfeeding
- Treatment with corticosteroids in dose >40 mg of prednisolone or > 9 mg of budesonide MMX
- History of bone marrow transplantation
- History of apheresis 12 months before randomization
- Fecal microbiota transplantation 8 weeks before screening
- History of HSV, HPV, influenza, or SARS-CoV2 infection 12 weeks before randomization or history of complicated HSC infection
- History of abdominal surgery: extensive colonic resection, subtotal or total colectomy, history of ileostomy, colostomy, or known fixed symptomatic stenosis of the intestine
- History or evidence of adenomatous colonic polyps that have not been removed or colonic mucosal dysplasia
- Evidence of abdominal abscess, toxic megacolon, and colon perforation 12 weeks prior to screening
- Usage of drugs is not allowed: methotrexate, cyclosporine, tacrolimus, sirolimus, tofacitinib, filgotinib, other JAK inhibitors, ozanimod, other biological therapies such as natalizumab, adalimumab, vedolizumab, golimumab
- Receiving any investigational or approved drugs from the list of drugs according to Table 1
- Any history of malignancy, except for the following: adequately treated nonmetastatic basal cell skin cancer, squamous cell skin cancer that has been adequately treated and that has not recurred for at least 1 year prior randomization, history of cervical carcinoma *in situ* that has been adequately treated and has not recurred for at least 3 years before randomization
- Evidence of *C. difficile* infection or other clinically significant intestinal pathogen infection at screening
- Any identified congenital or acquired immunodeficiency
- Any live vaccination within 30 days prior to screening or is planning to receive any live vaccination during participation in the study
- Any of the following laboratory abnormalities during the screening period:

**Table 1:** List of drugs not allowed in the study.

Drug/Therapy	Time of ending of the therapy before randomization	Drug/Therapy	Time of ending of the therapy before randomization
Abatacept (CTLA4Ig)	≥ 12 weeks	JAK inhibitors (tofacitinib, filgotinib, upadacitinib)	≥ 8 weeks
Adalimumab	≥ 8 weeks	Leflunomide	≥ 12 weeks
Alefacept	≥ 8 weeks	Memantine	≥ 4 weeks
Alemtuzumab	≥ 12 months	Methotrexate	≥ 4 t weeks
Belilumab	≥ 14 weeks	Mycophenolate mofetil	≥ 4 weeks
Certolizumab pegol	≥ 8 weeks	Natalizumab	≥ 8 weeks
Cyclofosfamide	≥ 4 weeks	Ozanimod	≥ 4 weeks
Cyclosporin	≥ 4 weeks	Pimecrolimus	≥ 4 weeks
Danazol	≥ 4 weeks i	Retinoids	≥ 4 weeks
Dapsone	≥ 4 weeks	Rituximab	≥ 12 months
Eculizumab	≥ 12 weeks	Sirolimus	≥ 4 weeks
Efalizumab	≥ 8 weeks	Tacrolimus	≥ 4 weeks
Epratuzumab	≥ 18 weeks	Talidomide	≥ 4 weeks
Golimumab	≥ 4 weeks	Tocilizumab	≥ 12 weeks
Interferon	≥ 12 weeks	Vedolizumab	≥ 8 weeks
Other investigational therapies	≥ 4 weeks or 5 half-life times before randomization	–

- ALT activity level > 3x upper limit normal (ULN)
- AST activity level > 3x ULN
- Total bilirubin level > 2x ULN (the exception is Gilbert’s syndrome when other isolated causes of hyperbilirubinemia are excluded)
- GGT or ALP activity ULN >3x GGN
- Creatinine level > 2x ULN or impaired renal function (eGFR) <45 mL/min calculated by the MDRD formula
- Hemoglobin level < 9 g/dL
- Leukocyte absolute number < 3,000/mm3
- Lymphocyte absolute number < 750/mm3
- Neutrophils level < 1,000/mm3
- Platelets level < 100,000/mm3

### 2.7 Trial procedures

**Figure 2:**
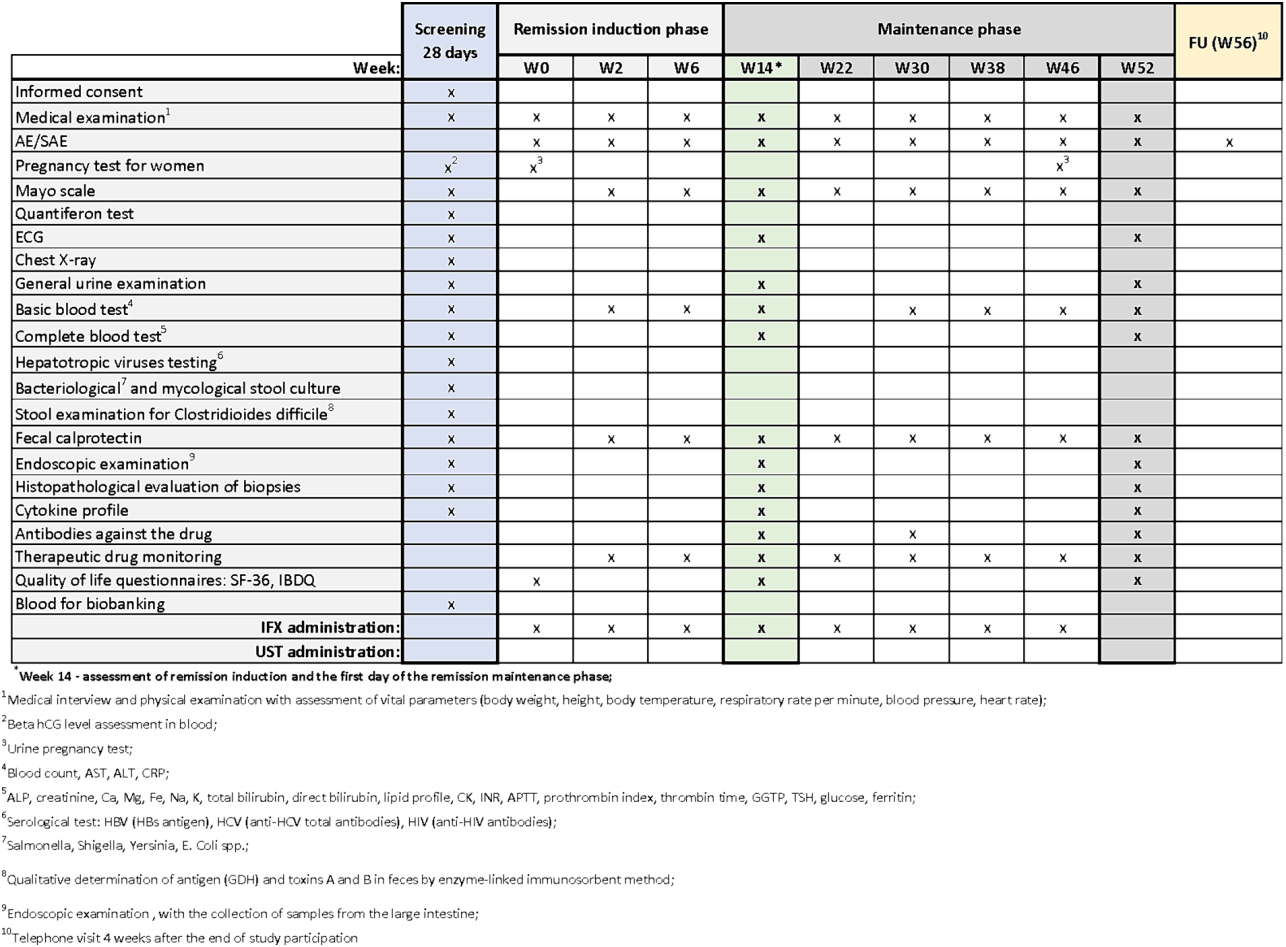
Scheme of medical procedures during screening, in the remission induction phase and maintenance phase in arm A.

**Figure 3:**
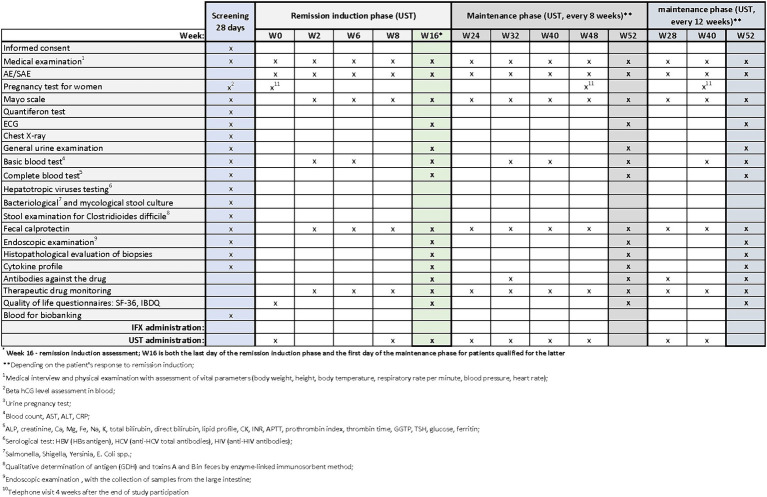
Scheme of medical procedures during screening, in the remission induction phase and maintenance phase in arm B.

**Figure 4:**
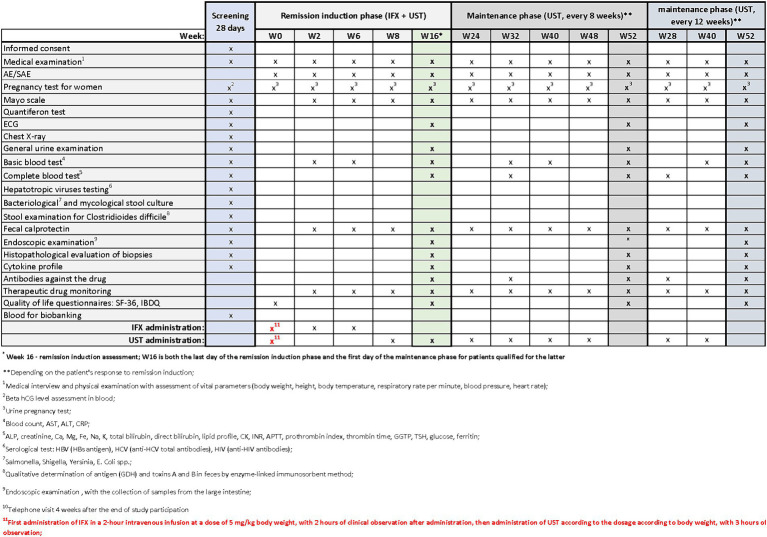
Scheme of medical procedures during screening, in the remission induction phase and maintenance phase in arm C.

Patients with UC who meet the clinical trial’s inclusion criteria may be enrolled in designated centers. The trial commences with a screening period, which will not exceed 28 days. During the screening visit, patients will receive comprehensive information about the study from the investigator. After addressing any questions related to the study and its protocol, the investigator will verify inclusion and exclusion criteria and obtain signed informed consent in duplicate. Upon successful recruitment, study procedures will commence. During the screening period, the procedures outlined in Figures 2–4 will be performed. These include a thorough medical history, a physical examination with assessment of vital signs, and the collection of biological samples (blood, stool, urine). Additionally, chest X-rays and endoscopy with colonic sample collection will be conducted. A full colonoscopy will be performed if the disease duration exceeds 8 years and the patient has not undergone a full colonoscopy within the last year; otherwise, sigmoidoscopy will suffice. Endoscopy procedures will be recorded using an external device, with patient data blinded, and the video sent for additional evaluation by a central blinded reader. The final decision regarding endoscopic disease activity will remain with the endoscopist performing the procedure.

Successful completion of the screening period will qualify the patient for the randomization visit (Week 0). During this visit, the patient will be randomized to one of the three study arms (A-B-C). Additionally, the patient will complete QoL questionnaires, including the 36-Item Short Form Health Survey (SF-36) and the Inflammatory Bowel Disease Questionnaire (IBDQ). Finally, the patient will receive the drug to which they were randomized starting the remission induction phase.

The next visits in the remission induction phase will take place at Weeks 2 and 6 in arm A, and at Weeks 2, 6 and 8 in arms B and C. The assessment of remission induction in arm A will take place at Week 14. If the patient is positively qualified for the remission maintenance phase, the first day of remission maintenance will be considered. In case of arms B and C, the assessment of remission induction will take place at Week 16. This week will also be the first day of maintenance of remission after the patient is successfully qualified for maintenance. Subsequent visits are scheduled as follows:

- Arm A: Weeks 22, 30, 38, 46
- Arms B and C: Weeks 24, 32, 40, 48 (with UST administered once every 8 weeks) or Weeks 28 and 40(with UST administered once every 12 weeks).

In all arms, starting from Week 2 and on each subsequent visit, patients will undergo physical examination with vital signs assessment and evaluation of the Mayo score. AEs and SAEs will be recorded, and basic blood parameters, calprotectin levels, and drug levels will be assessed. The end-of-study (EoS) visit will occur at Week 52. Additionally, ECG and endoscopy with colonic sampling will be performed, and patients will also complete the QoL questionnaires. In arm A, extended blood samples will be taken at Week 30, while in arms B and C, this will occur at Week 32 (with UST administered once every 8 weeks) and at Week 28 (with UST administered once every 12 weeks). Drug antibody evaluation will be conducted during these visits. In experimental arm C, female patients will undergo urine pregnancy tests at each visit from the screening visit up to Week 52.

The study will conclude with a telephone follow-up visit at Week 56, during which AE/SAE evaluations will be conducted.

### 2.8 Tools and parameters used during the trial

During visits, standard procedures will be performed in accordance with the medical procedures (Figures 2–4). Patients’ quality of life will be measured using two quality of life questionnaires IBDQ and SF-36 (18).

The questionnaires were translated into Polish and purchased for the study along with a license enabling the use of the questionnaires for the study. The IBDQ divides quality of life into 4 dimensions: gastrointestinal symptoms, systemic functioning, emotional functioning, and social functioning. A meta-analysis conducted in 2020 showed that the IBDQ exhibits a high correlation between QoL in patients and treatment response in individuals with IBD (23). However, incorporating an additional questionnaire not only enhances the comprehensiveness of the assessment but also expands the scope of the evaluated QoL aspects. The SF-36 evaluates various factors including limitations related to physical health, the influence of physical functioning on daily activities, pain levels, overall perception of health, the influence of emotional well-being on daily functioning, social interactions, and vitality. Therefore, following the established standards in international clinical trials with IBD, we decided to use the two mentioned questionnaires (IBDQ and SF-36), considering their confirmed effectiveness in research applications and simultaneous good patient acceptance during completion.

**Figure 5:**
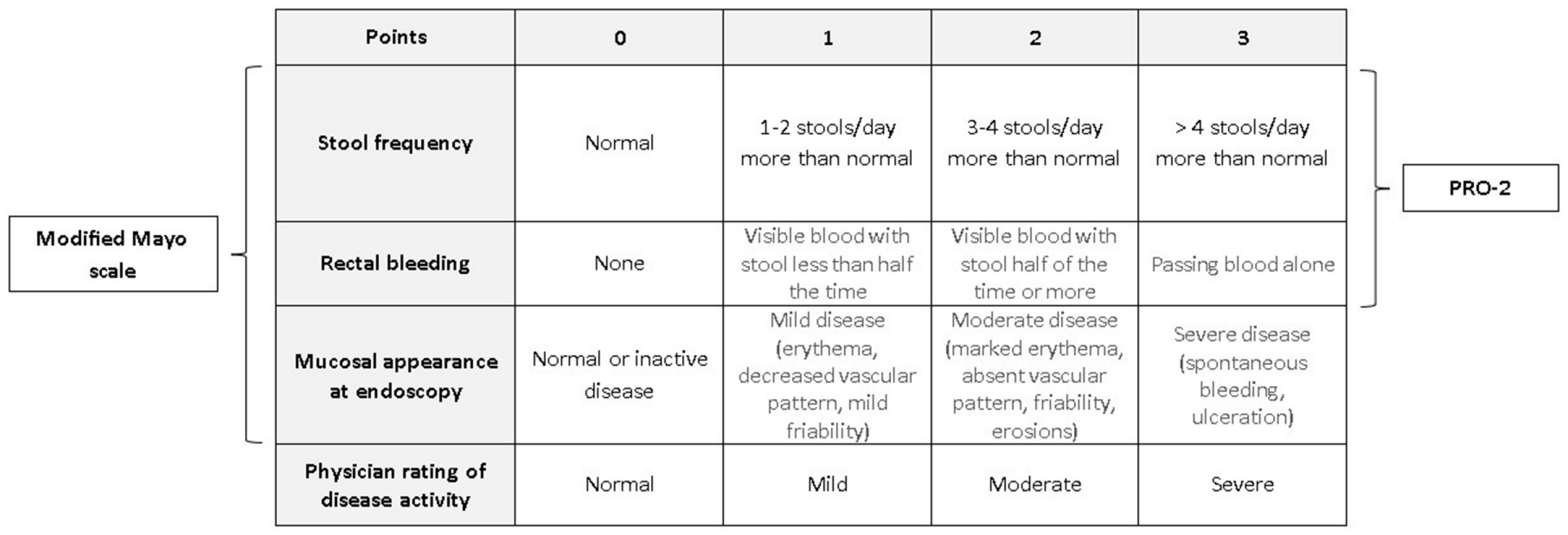
Total Mayo score with its subtypes: modified Mayo score and PRO-2.

For assessing disease activity, the Mayo Score will be employed (Figure 5). Among the numerous disease activity indices, the Mayo Score has been widely accepted and is most commonly used in clinical trials and clinical practice in adults. Furthermore, the definitions of clinical remission and clinical response to therapy will be correlated with the improvement in patients’ QoL as assessed by IBDQ and SF-36.

### 2.9 Randomization

Patients will be randomly assigned using block randomization stratified by the trial center in a 1:1:2 ratio between arms A, B and C, respectively. Randomization will be performed using the “Randomizer” IT tool provided by the study Sponsor, integrated with electronic case report forms (eCRF). In the event of difficulties with randomization, such as lack of Internet access or other technical problems, it will be possible for the study coordinator at a given center to request randomization via the backup randomization list available from the Sponsor’s representative, and it will also be possible to re-randomize or withdraw the patient.

### 2.10 Blinding

Due to the comparative nature of the study, aiming to compare the effectiveness of dual biological therapy with biologic drugs administered solely, we have not implemented treatment blinding. However, we have included a blinded central endoscopy reader. Each endoscopy procedure will be recorded using an external device and sent to the central reader who will be blinded to the patient’s treatment arm. It is important to note that the final decision regarding the endoscopic disease activity will remain with the endoscopist performing the procedure.

### 2.11 Endpoints

The primary endpoint will be the percentage of patients with clinical and endoscopic remission after the induction phase (Week 14 for arm A and Week 16 for B and C).

The secondary endpoints will encompass:

- Percentage of patients with clinical response after the induction phase (Week 14 for arm A and Week 16 for B and C).
- Percentage of patients with clinical response at Week 52.
- Percentage of patients with clinical remission at Week 52.
- Percentage of patients with endoscopic response or remission after the induction phase (Week 14 for arm A and Week 16 for B and C) and at Week 52.
- Percentage of patients with biochemical remission after the induction phase (Week 14 for arm A and Week 16 for B and C) and at Week 52.
- Percentage of patients with histological remission after the induction phase (Week 14 for arm A and Week 16 for B and C)
- Comparison of patients’ QoL after the induction phase (Week 14 for arm A and Week 16 for B and C) and at Week 52 using SF-36 and IBDQ indices.
- Safety analyses, including AEs and SAEs, after the induction phase (Week 14 for arm A and Week 16 for B and C) and at Week 52.

Definitions:

- Clinical remission: PRO-2 score ≤ 1 points (24), total Mayo score < 3 (25)
- Endoscopic remission: endoscopic Mayo score ≤ 1
- Clinical response: reduction in PRO-2 score ≥ 50% (1) or a reduction in total Mayo score ≥ 3 points (25)
- Endoscopic response: reduction in endoscopic Mayo score ≥ 1 point
- Biochemical remission: calprotectin level ≤ 125 μg/g of stool
- Deep remission: PRO-2 score = 0 points, endoscopic Mayo score = 0 points and Picasso Histologic Remission Index = 0 points

### 2.12 Statistical analysis

The primary endpoint described as difference in frequency clinical remission was defined as PRO-2 decrease <2 and decrease in the endoscopic Mayo≤1 score and also as the Mayo full scale score < 3, will be measured after screening and randomization in the week 14 and 16 which will end remission induction phase. Analysis of differences in the incidence of the primary endpoint will be performed between the IFX + UST combined arm and the IFX and UST monotherapy arms. The incidence of achieving the composite endpoint will be compared between the IFX + UST study group in arm C and the control group presented with IFX and UST monotherapy in arms A and B using the Pearson *Χ*^2^ test.

To assess the key secondary endpoint of clinical response rate after the remission induction phase at week 14 and 16 (defined as a decrease in PRO-2 of at least 50% and a decrease in the endoscopic Mayo score ≥ 1 and a decrease in the total Mayo score ≥ 3 points), the proportion of clinical response in the form of a composite endpoint will be compared between the study group in arm C (IFX + UST) and the control group in arm A + B (IFX and UST) using the Pearson chi-square test. In the case of other secondary endpoints, analyses will be performed analogously in the form of proportions between the study group in arm C (IFX + UST) and the control group in arms A and B (IFX and UST) using Pearson’s Χ^2^ tests.

The quality of life of patients will be assessed using the SF-36 and IBDQ. The points obtained by an individual patient will be calculated according to validated procedures attached to the manual and/or instructions for the questionnaires.

For the purpose of exploratory analysis, statistical analysis will be performed using classification models based on logistic regression techniques, decision trees, ROC curves and discriminant analyses, aimed at identifying predictive indicators for the success or failure of therapy and the occurrence of side effects, based on measurements of the concentrations of cytokines associated with the presence of the disease and the severity of the disease process.

Safety analysis will be conducted using “As-Treated” (AT) and will be contained AE and SAE reported and other critical information. All events will be summarized in relation to treatment arm. For each variable and treatment group, the number and percentage of patients who experienced treatment-related adverse events, including those requiring treatment discontinuation, will be presented. Treatment safety will be assessed and reported following standard procedures – coded using MeDRA V.24.1. All information about safety will be recorded in eCRF.

### 2.13 Missing data

For missing data on primary or secondary endpoints for patients in the study, these data will not be completed and will be treated as observations truncated at the time of the last observation. The truncated observations will be used for exploratory and security analysis.

For missing data at the last observation or values for nominal variables at visits in weeks 2–48, the Last Observation Carried Forward (LOCF) imputation method will be used, replacing the missing value with the last observed value from the same facility. Missing values of two or more values will not be corrected during consecutive visits.

Missing data in other variables will be filled based on adjacent visits. For continuous variables, the average value calculated from the preceding and subsequent observations will be adopted. Statistical analysis will be performed based on full data with imputation, and sensitivity analysis will be performed after excluding imputed values. A detailed description of the statistical methods will be included in the statistical analysis plan.

### 2.14 Sample size estimation

The sample size was calculated based on the randomized, double-blind phase 2a VEGA study (15) which was presented for ECCO congress in 2022. The study compared patients with ulcerative colitis (UC) who received remission induction treatment in combination therapy monoclonal antibodies guselkumab (GUS) and golimumab (GOL) versus patients was using monotherapy GUS or GOL. Clinical remission and endoscopic improvement were more often obtained by patients treated with the combination therapy (36.6 and 49.3% respectively) compared with monotherapy GUS (21.1 and 29.6%) and GOL (22.2 and 25%). The rate of histological remission in this study was 26% (GUS) and 15% (GOL) with a 41% response achieved in the combined GUS + GOL arm. In the COMBO-UC study we expect that remission rates in A and B-arm reach 20% and at least 40% in the C-armTo confirm significance at an alpha level for 0.05 for an effect of such magnitude or larger in a Chi2 test with 80% statistical power one would require 164 patients. Taking into account the potential benefits for patients and the importance of the experimental arm compared to available alternative therapy methods, the allocation to arms A, B and C was planned to be 1:1:2, respectively, i.e., 41, 41 and 82 patients. This number was increased to 172 patients total (43, 43, 86) to take into account the possible drop-out of patients from the study at the 5% level. A low drop-out rate was assumed due to the limited selection of alternative therapies and the strong impact of uncontrolled disease on the quality of life as well as the observed frequency of complications forcing the cessation of therapy under previously used drug programs in arms A and B of the study. The size of the groups will allow for the demonstration of statistical significance for differences in the effectiveness of arms A/B and C expressed as secondary endpoints, greater than RR < 0.5 or RR > 2 for complications during the treatment maintenance period. For the purposes of exploratory analyses, the number of 86 patients in arm C will allow, assuming a 40–50% clinical remission rate, the identification of biomarkers predictive of the effectiveness of combined treatment with AUROC >0.75 (with a power of 80% I at α = 0.05) taking into account a 10% dropout before finishing treatment. Sample size and statistical power analysis was performed using PASS 2020 Power Analysis and Sample Size Software (2020) version 20.0.10. NCSS, LLC. Kaysville, Utah, United States, ncss.com/software/pass.

### 2.15 Trial monitoring

Monitoring will be performed by Sponsor or contracted to a quality CRO. Monitoring will include on-site, remote and risk-based monitoring. The monitoring plan will contain monitoring details describing strategy, including study-critical data and processes, (e.g., risk management and risk-based monitoring), methods, responsibilities, requirements including monitoring techniques (central, remote and on-site). Monitors will contact and/or visit site before and during study.

The purpose of the visits will be to verify that safety and rights of participants are being protected, assess to progress of the study, review the compliance with approved protocol, ICH GCP and all applicable regulatory requirements and source data verification and source data review.

The inspection may be carried out at the trial site, at the sponsor’s or at the site of the organization responsible for conducting the contracted clinical trial (CRO) or at another site deemed appropriate by the relevant authorities.

Representatives of national regulatory authorities may also evaluate study documentation, source documents, the investigator, study personnel and facilities.

### 2.16 Trial timeline

The planned date for starting the patient recruitment is the third quarter of 2024 but may vary due to logistic reasons. The trial is planned to last 48 months. We expect to complete the study by the end of 2028.

### 2.17 Ethical considerations

The clinical trial will be conducted in accordance with applicable legal and ethical standards.

The clinical trial protocol and its amendments, if any, as well as the Patient Information Form and the Informed Consent Form were subject to review by the Independent Bioethics Committee and the President of the Office for Registration of Medicinal Products, Medical Devices and Biocidal Products. The study was registered under EU CT number 2023–506452–25-00 and received approval on April 12, 2024. All changes to the study will be subject to ethical and regulatory review before being put into practice.

### 2.18 Dissemination plan

Results will be submitted for publication in leading international scientific journals in gastroenterology. Additionally, findings will be presented at relevant national and international congresses and conferences. The study’s results will also be communicated to the funding agency and national healthcare policymakers.

## 3 Conclusion

To conclude, it must be emphasized that recent advances in pharmacological treatment options in UC have significantly changed short-and long-term prognosis. The increasing number of targeted therapeutic molecules allow achieving more ambitious goals like mucosal and histological healing of the inflamed colonic tissues. However, in spite of these advances, still a significant proportion of patients do not respond to the therapy. To address this unmet need of improving treatment outcomes in UC, a novel approach of applying DTT has been proposed.

The COMBO-UC Study, which is planned to begin enrolling patients in Poland in 2024, will verify whether combining two different modes of action by using UST and IFX can be beneficial for patients with moderately-to-severely active UC. We believe that the results of this randomized trial will be helpful in changing treatment algorithms in UC in the near future.
